# Supplementary material for: Chronically Low NMNAT2 Expression Causes Sub-lethal SARM1 Activation and Altered Response to Nicotinamide Riboside in Axons
Source: Mol Neurobiol. 2024 Oct 1;62(3):3903–17. doi: 10.1007/s12035-024-04480-2 (PMC11790816; doi:10.1007/s12035-024-04480-2)

Chronically low NMNAT2 expression causes sub-lethal SARM1 activation and altered response to nicotinamide riboside in axons

Molecular Neurobiology

Christina Antoniou[1], Andrea Loreto[1,3], Jonathan Gilley[1], Elisa Merlini[1], Giuseppe Orsomando[2] and Michael P Coleman[1]

Corresponding author: Michael Coleman mc469@cam.ac.uk

Author affiliations:

1. John van Geest Centre for Brain Repair, Department of Clinical Neurosciences, University of Cambridge, Forvie Site, Robinson Way, CB2 0PY Cambridge, UK
2. Department of Clinical Sciences (DISCO), Section of Biochemistry, Polytechnic University of Marche, Via Ranieri 67, Ancona 60131, Italy
3. School of Medical Sciences, Charles Perkins Centre, Faculty of Medicine and Health, The University of Sydney, Sydney, NSW, Australia

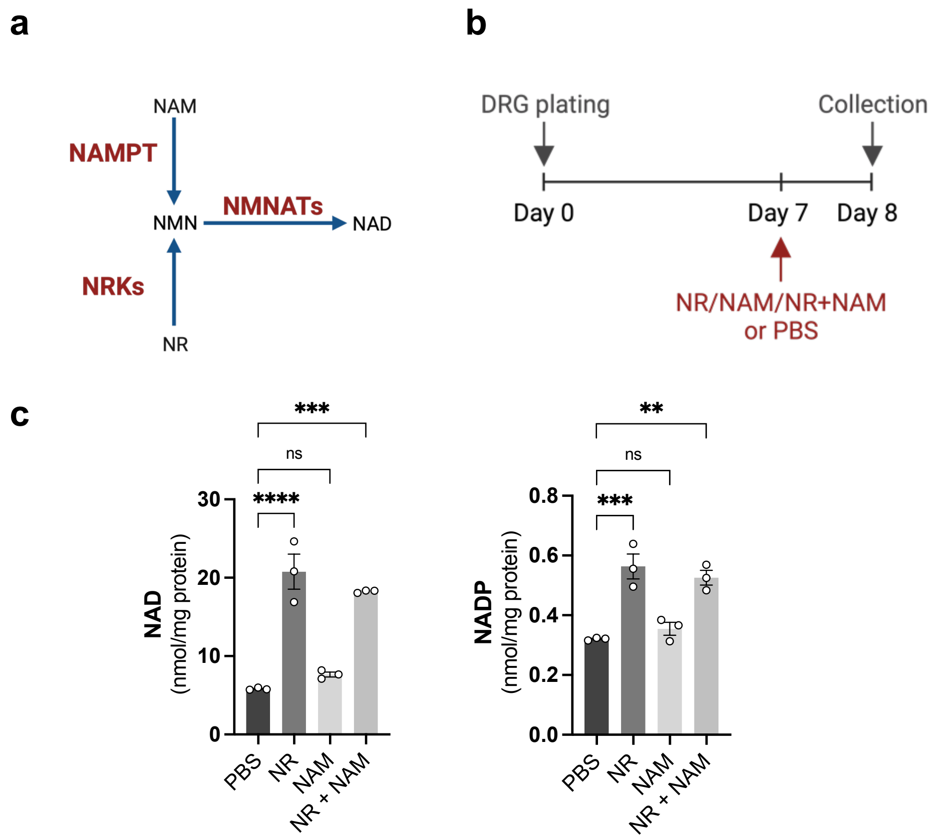

Supplementary figure 1 Testing supplementation of NAD precursors in primary neuronal cultures. (**a**) Pathway of NAD synthesis from precursors NAM (nicotinamide) and NR (nicotinamide riboside). (**b**) Timeline of NAD precursor administration and collection of whole DRG cultures. (**c**) NAD and NADP levels in wild-type DRG explants following administration of NR (2 mM), NAM (1 mM), combination of NR and NAM or PBS control (mean ± SEM; n = 3; ****p < 0.0001, ***p < 0.001, **p < 0.01 and ns (not significant) = p > 0.05, one-way ANOVA with Dunnett's test comparing means to PBS control).

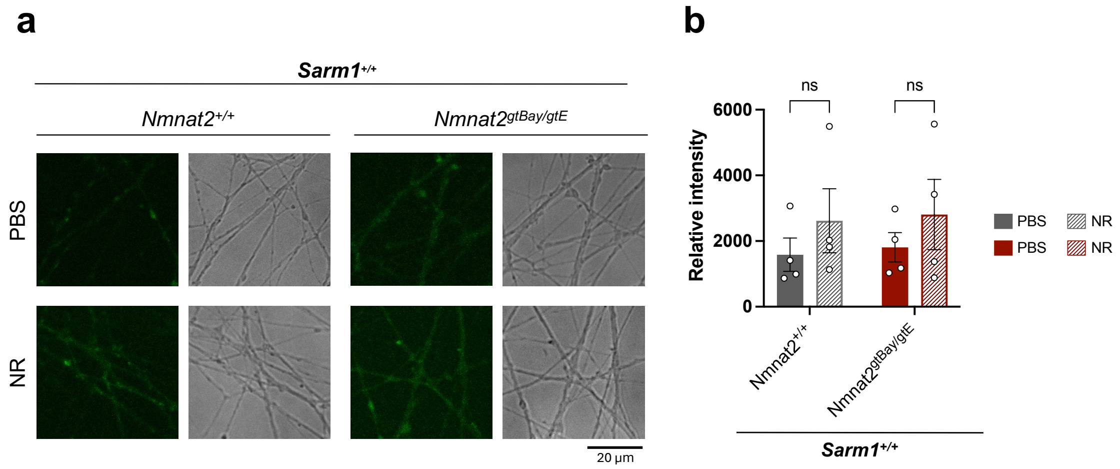

Supplementary figure 2 Testing SARM1 activation in neurites following administration of NR. (**a**) Representative images of *Nmnat2*$^{+/+}$ and *Nmnat2*$^{gtBay/gtE}$ SCG neurons on a *Sarm1*$^{+/+}$ background treated with NR (2mM) or PBS control, 30 min after incubation with PC6 (50 µM). (**b**) Quantification of fluorescence intensity of PAD6 in *Nmnat2*$^{+/+}$ and *Nmnat2*$^{gtBay/gtE}$ SCG neurites on a *Sarm1*$^{+/+}$ background treated with NR (2mM) or PBS control (mean ± SEM; n = 4; ns (not significant) = $p > 0.05$, multiple paired t-tests for PBS vs NR with Holm-Šídák correction method).

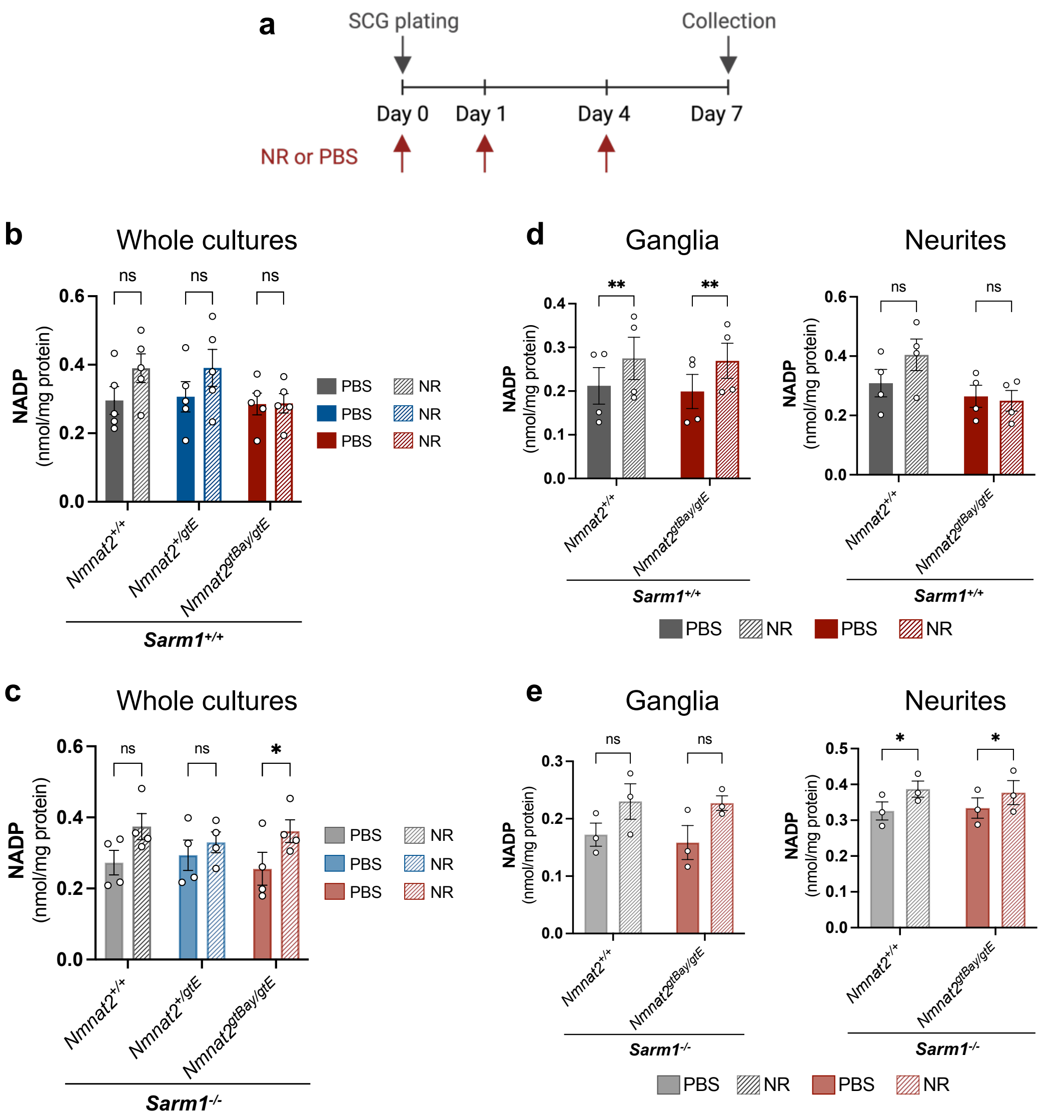

Supplementary figure 3 NR administration does not increase NADP levels in SCG whole cultures and neurites from *Nmnat2*$^{gtBay/gtE}$ mice. (**a**) Timeline of NR (2 mM) or PBS administration and collection of SCG cultures. (**b**) NADP levels in SCG explants of the indicated genotypes, all on a *Sarm1*$^{+/+}$ background (mean ± SEM; n = 5; ns (not significant) = p > 0.05, multiple paired t-tests for PBS vs NR with Holm-Šídák correction method). (**c**) NADP levels in whole SCG explants of the indicated genotypes, all on a *Sarm1*$^{-/-}$ background (mean ± SEM; n = 4; *p < 0.05 and ns (not significant) = p > 0.05 multiple paired t-tests for PBS vs NR with Holm-Šídák correction method). (**d**) NADP levels in SCG ganglia and neurites of the indicated genotypes, all on a *Sarm1*$^{+/+}$ background (mean ± SEM; n = 4; **p < 0.01 and ns (not significant) = p > 0.05, multiple paired t-tests for PBS vs NR with Holm-Šídák correction method). (**e**) NADP levels in SCG ganglia and neurites of the indicated genotypes, all on a *Sarm1*$^{-/-}$ background (mean ± SEM; n = 3; *p < 0.05 and ns (not significant) = p > 0.05, multiple paired t-tests for PBS vs NR with Holm-Šídák correction method).

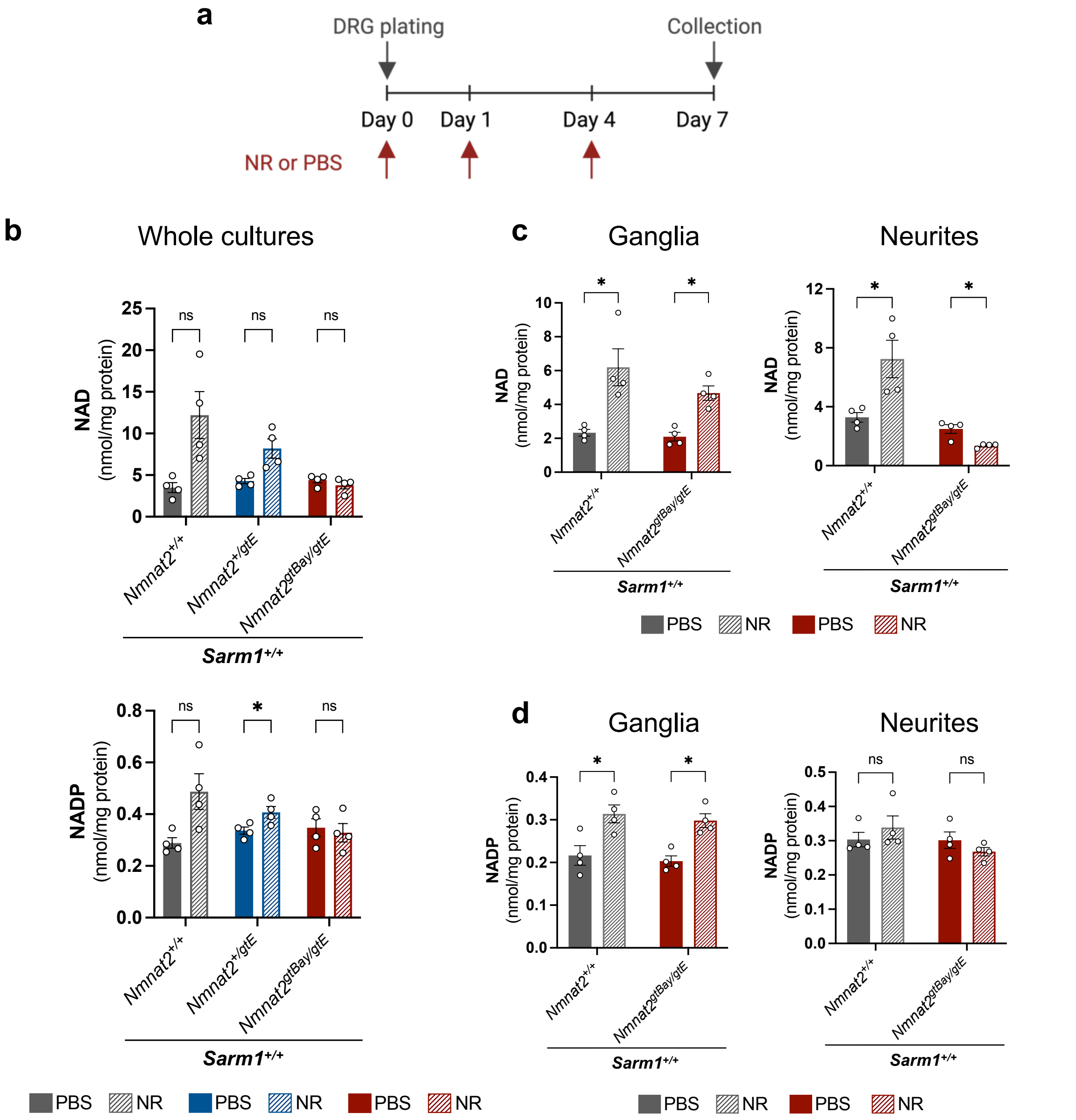

Supplementary figure 4 NR causes an NAD depletion in DRG neurites from Nmnat2$^{gtBay/gtE}$ mice. (**a**) Timeline of NR (2 mM) or PBS administration and collection of DRG cultures. (**b**) NAD and NADP levels in whole DRG explants of the indicated genotypes, all on a *Sarm1*$^{+/+}$ background (mean ± SEM; n = 4; *p < 0.05 and ns (not significant) = p > 0.05, multiple paired t-tests for PBS vs NR with Holm-Šídák correction method). (**c**) NAD levels in DRG ganglia and neurites of the indicated genotypes all on a *Sarm1*$^{+/+}$ background (mean ± SEM; n = 4; *p < 0.05 multiple paired t-tests for PBS vs NR with Holm-Šídák correction method). (**d**) NADP levels in DRG ganglia and neurites of the indicated genotypes, all on a *Sarm1*$^{+/+}$ background (mean ± SEM; n = 4; *p < 0.05 and ns (not significant) = p > 0.05, multiple paired t-tests for PBS vs NR with Holm-Šídák correction method).

Uncropped immunoblot images for figure 2e

Membrane probed for SARM1

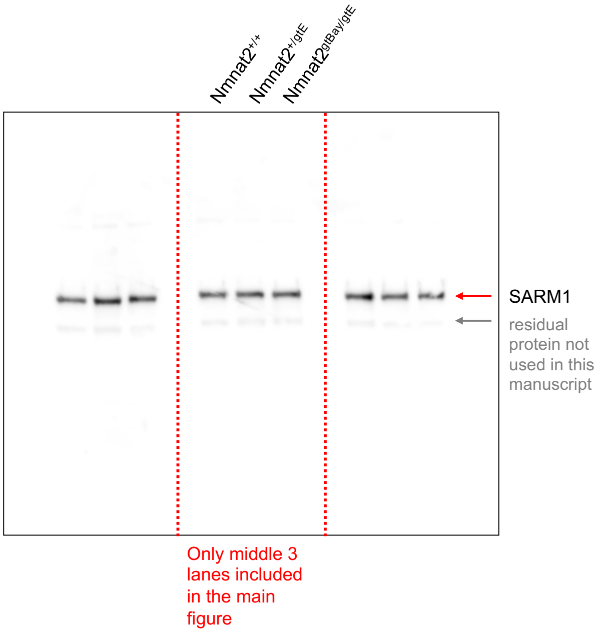

Membrane probed for GAPDH

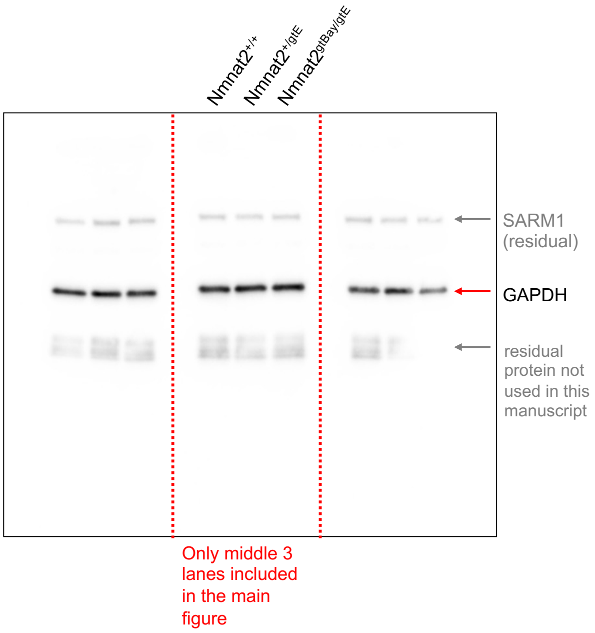

Uncropped immunoblot images for figure 4b

Membrane probed simultaneously for SARM1 and NAMPT

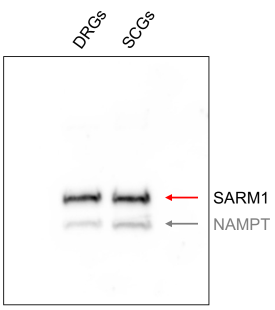

shorter exposure for SARM1 quantification

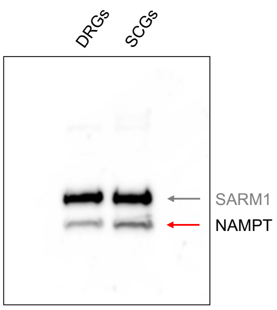

longer exposure for NAMPT quantification

Membrane probed for NMNAT2

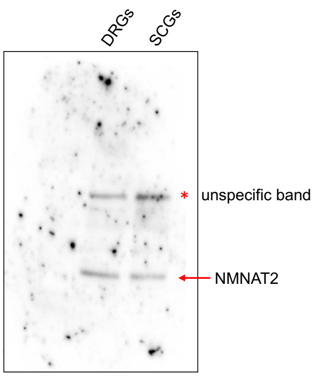

Membrane probed for GAPDH

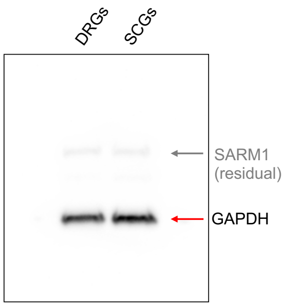

Supplement: Supplementary file 1 — Supplementary file1 (PDF 1.37 MB) [file 12035_2024_4480_MOESM1_ESM.pdf]
